# Effect of hydroalcoholic extract of flaxseed on bone mineral density in Wistar rats using digital radiography

**DOI:** 10.22088/cjim.11.1.92

**Published:** 2020

**Authors:** Farideh Nozari Moshtaghin, Ali Akbar Moghadamnia, Sohrab Kazemi, Nazanin Arbabzadegan, Ehsan Moudi, Sina Haghanifar

**Affiliations:** 1 Student Research Committee, Babol University of Medical Sciences, Babol, Iran; 2 Neuroscience Research Center, Health Research Institute, Babol University of Medical Sciences, Babol, Iran; 3 Cellular and Molecular Biology Research Center, Health Research Institute, Babol University of Medical Sciences, Babol, Iran; 4 Oral Health Researche Center, Health Research Institute, Babol University of Medical Sciences, Babol, Iran

**Keywords:** Bone Mineral density, Flaxseed, Digital radiography, Rat

## Abstract

**Background:**

Given that the world's population is aging, the problems associated with osteoporosis and related fractures are increasing. The aim of the study was to evaluate the effect of flaxseed extract on bone mineral density (BMD) in Wistar rats using digital radiography.

**Methods:**

In this experimental study, 25 male and 25 female Wistar rats were randomly divided into 5 groups: 1. Control, 2. Calcium and vitamin D (Ca/VitD), 3. 100 mg/kg flaxseed, 4. 200 mg/kg flaxseed, and 5. 400 mg/kg flaxseed. Then, the animals were kept for thirty days. Maxillary and mandibular BMD as well as serum levels of calcium, vitamin D and phosphorus were measured at baseline and after 30 days of keeping the rats.

**Results:**

The results showed that serum levels of calcium and phosphorus were not significantly different in all five groups before and after 30 days. Serum levels of vitamin D were significantly higher in the group receiving Ca/vit D (with a mean of 61.6±15.8 in the male group and 85±12.9 in the female group) as compared with other groups (P<0.001). The highest level of change in maxillary and mandibular bone density was in 200 mg/kg flaxseed group with a mean difference of 24.5±6.1 and 26.5±3.1, respectively, which was significant in comparison with the control and Ca/vit D groups (p<0.001) .

**Conclusion:**

Flaxseed extract is more effective in increasing bone density than the group receiving Ca/vit D. The mandibular and maxillary BMD was higher in the group receiving 200 mg/kg flaxseed compared to the group receiving Ca/vit D (p<0.001).

Achieving peak bone mass is necessary to attain optimal bone health and hence, reducing the risk of osteoporosis in the future (1). Achieving peak bone mass will continue until late adolescence or early 20s (2), depending on interactions between hormones and growth factors, genetics, physical activity as well as nutrition, particularly Ca/VitD (3-5). The role of other specific dietary compounds such as phytoestrogens, estrogens and anti-estrogenic agents remains unclear for achieving peak bone mass (6). Substances that are present in living organisms such as animals and plants and acting like estrogens are called peripheral estrogens. Some of these hormones called phytoestrogens are found in plants such as soy clover and flaxseed. These hormones are herbal compounds that structurally and functionally act like estrogen (7). Flaxseed (Linum usitatissimum L) contains a high amount of phytoestrogens. Flaxseed phytoestrogen is called secoisolariciresinol diglucoside (SDG) lignan, which becomes enterolactone (EL) and enterodiol (ED) in the intestine (8). The structure of these two substances is similar to 17β-estradiol, which has both estrogenic and anti-estrogenic activities. It differs depending on the dosage, the duration of use and the stage of consumer’s development (9-12). 

The clinical effects of phytoestrogens, as weak estrogen agonists, are well demonstrated in menopausal syndrome. When the estrogen levels are low in the environment, the estrogenic effects become more powerful; therefore, the prediction that these substances can provide more estrogenic properties in postmenopausal women may be true (13-15). 

Recently, it has shown that the aromatization reaction of androgens to estrogens is necessary for both growth and maintenance of the skeletal integrity (16-18). It has been indicated that enterolactone (EL) has a moderate inhibitory activity on aromatase and occurs to bind to the active site of the P-450 enzyme by competing with testosterone. Therefore, it is not yet clear whether consumption of lignan-rich foods with potential estrogen-like activity can reduce bone development (by reducing aromatase activity), or it has other hormonal and non-hormonal mechanisms (19). In addition, flaxseed (Linum usitatissimum L) is known as an excellent source of alpha-linolenic acid (ALA, 18: 3n-3) (20). ALA plays an important role in maintaining or increasing bone mass by increasing the differentiation of mesenchymal stem cells into osteoblasts (21).

Moreover, ALA enhances the absorption of calcium from the intestine and accelerates the deposition of calcium into the bone (22). On the other hand, the protein in the flaxseed leads to organic matrix deposition, including a specific protein of the bone such as type I collagen increases bone strength and resistance (23). Flaxseed oil is associated with increase in low-density lipoprotein (LDL) and decrease in cholesterol. Experimental studies have shown that cholesterol directly interferes with osteoblastic differentiation by reducing bone formation and increasing osteoclast activity and there is a positive correlation between LDL and bone mineral density (BMD) (24, 25).

In a study by Ward et al. on female (26) and male (27) rats using flax lignans evaluated the effect of flax lignans on femoral bone of rat using DEXA (dual-energy x-ray absorptiometry). This study investigated the effect of flaxseed extract on jaw bone density via digital radiography. Densitometry can be performed to measure BMD, which is done in different ways (28-31). One of the ways is the use of digital radiography, which provides different tools such as digital ruler and densitometer. Therefore, digital radiography is a good option for determining bone density since it is inexpensive, easily accessible, and is commonly used (32, 33). In some studies, digital radiography has been used to measure bone density (34, 35). The aim of this study was to evaluate the effect of flaxseed extract on BMD using digital radiography as well as serum levels of calcium, phosphorus and vit D.

## Methods

This experimental study was approved by Babol University of Medical Sciences Ethics Committee (code no: MUBABOL.HRI.REC.1396.194). The hydroalcoholic extract was prepared after providing, washing, drying and grinding flaxseed. The solutions were obtained from the extract at doses of 100, 200 and 400 mg/kg body weight. Four-week-old Wistar rats (100 grams) were used in this study. All rats were divided into two groups based on gender. Then, the male rats were divided into 5 groups as follows: 

Group 1: Control group with daily nutrition.

Group 2: Daily nutrition with 200 mg/kg body weight Ca/VitD

Group 3: Daily nutrition with 100 mg/kg flaxseed extractGroup 4: Daily nutrition with 200 mg/kg flaxseed extractGroup 5: Daily nutrition with 400 mg/kg flaxseed extract.

**Figure 1:**
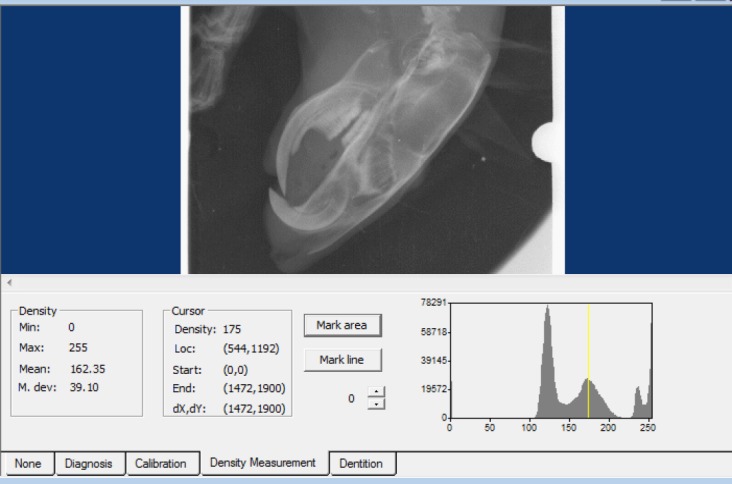
BMD measurement using Digora for Windows (DfW 2.7)

Selection of these dose for flaxseed extract is based on this reference (36). Similar grouping was designed for female rats. The rats received treatment by gavage. First, blood samples were collected from all rats and the calcium, phosphorus and vitamin D levels were measured by ELISA method (IDS company for vitamin D and Pars Azmoon for calcium and phosphorus). Then, the rats were anesthetized by intraperitoneal injection of a combination of 2 ml ketamine (100 mg/ml), and 1 ml xylazine (20 mg/ml), and the density of the samples was measured by digital radiography. Radiographs were taken using Pro X digital dental imaging device (Planmeca-Helsinki, Finland) under identical conditions in terms of position, distance, peak voltage (kVp), amperage (mA) and time. For the *radiographs* to be *taken*, the anesthetized rats were placed in a pre-prepared box and radiography was performed under the following conditions: kVp=50, mA=8, time=0.50 s, and distance from the X-ray tube: x=50 cm. Radiographs of the rat's head were taken using digital *phosphor* storage plate (PSP) (Soredex - Helsinki, Finland) sensors (size 4). Then, PSPs were processed by PCT (Soredex - Helsinki, Finland), and spongy bone density was measured at five points of each jaw using Digora for Windows (DfW 2.7) and the obtained average value was recorded (figure 1). 

Then, each group was kept at a temperature of 24 °C for 30 days under a 12:12 h light-dark cycle in a room with the same conditions and separate stainless steel cages with standard size. The first group had the usual daily diet. The second group had Ca/vit D in addition to the daily diet. Groups 3, 4, and 5 also received flaxseed extract by gavage. After 30 days, blood samples were collected again and the calcium, phosphorus and vitamin D levels were measured using ELISA kit. The rats were then anesthetized and their BMD was measured by radiography under similar conditions. Finally, the data were analyzed using SPSS Version 17 for windows and ANOVA. After performing ANOVA, Tukey's posthoc test was used. A P<0.05 was considered a statistically significant level for the difference between groups.

## Results

Maxillary and mandibular BMD as well as serum levels of calcium, vitamin D and phosphorus at baseline and after 30 days of maintaining ten groups of rats (5 rats in each group) are illustrated in table 1.

**Table 1:** Mean levels of BMD and serum calcium, vitamin D and phosphorus levels before and after 30 days, based on gender

		**Calcium**		**Phosphorus**		**Vitamin D**		**MAX.BMD** ^1^		**MAN.BMD** ^2^	
		**Before**	**After**	**Before**	**After**	**Before**	**After**	**Before**	**After**	**Before**	**After**
Control*	male	8.8±0.9	9±0.3	9.42±0.5	7.57±0.9	25.8±13	24.7±5.8	173.1±5.5	177±6.7	189.2±3.3	196.8±2.6
	female	9.76±0.3	8.86±0.29	10.3±0.49	7.04±0.65	28.06±1.8	29.5±1.9	171.5±2.4	168±3.8	188.2±1.7	188±2.1
VitD/Ca*	male	9.9±0.27	8.9±0.10	10.8±0.65	7.35±0.4	18.4±3.2	61.6±15.8	170.1±2.6	175.2±2.9	185.2±2.8	198.8±2.1
	female	9.88±0.3	8.95±0.12	9.18±0.9	6.42±0.35	29.7±6.9	85±12.9	174.6±6.4	174.04±2	188.1±3.1	195±3.9
Flax** 100	male	9.26±0.48	8.82±0.19	7.9±0.98	7.36±1.1	20.84±2.4	36.2±5.1	166.16±3	177.68±4.5	185.4±3.3	200.7±2.1
	female	9.38±0.66	8.48±0.34	10.4±0.47	7.22±0.92	30.2±2.6	33.02±4.3	160.88±1.9	182.3±6.3	174.9±3.3	199.6±3.6
Flax 200	male	9.64±0.4	9.2±0.2	9.4±2	8.8±0.6	23.3±5.1	26.26±1.7	161.3±1.4	181.7±3.3	181.85±1.9	204±2.3
	female	9.18±0.4	8.97±0.26	9.84±1.2	8.27±1.1	25.14±2.9	33.35±4.7	150.28±17.7	178.96±5.5	169.16±15.5	199.8±5.2
Flax 400	male	9.1±0.33	9.12±1.7	8.28±1	6.45±0.44	21.78±4.6	27.95±6.4	169±2.1	181.3±1.1	187±0.28	201.56±2.3
	female	9.28±0.86	8.7±0.15	9.56±0.93	7.6±1.01	31.9±9.04	31.9±6	168.4±4.2	185±6.7	185.48±5.1	204±5.3

1: maxillary BMD, 2: mandibular BMD *: These groups are shared with research project (code no: 9644814) at Babol University of Medical Sciences **: Flaxseed extract

**Figure 2:**
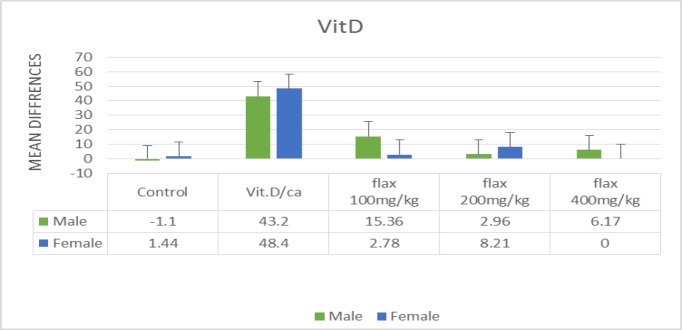
Comparison of the mean difference of serum vitamin D level based on gender

The results indicated that the serum calcium and phosphorus levels and their mean difference did not significantly change in any group after 30 days and there was no significant difference between males and females. The mean difference of serum vitamin D was significantly higher in the group receiving Ca/vit D compared to the control group and the groups receiving flaxseed extract in both genders (p<0.001) (figure 2). In terms of maxillary BMD, the density increased in all of the three groups receiving flaxseed compared to the control group and the group receiving Ca/vit D. The mean difference of this increase was significant in the group receiving 200 mg/kg flaxseed compared to the control group and the group receiving Ca/vit D, but was not significant in the groups receiving 100 and 400 mg/kg flaxseed compared to the control group and the group receiving Ca/vit D (table 2). The mandibular BMD was significantly higher in the group receiving 200 mg/kg flaxseed compared to the other four groups and this increase was significant compared to the control group, the group receiving Ca/vit D, and the group receiving 400 mg/kg flaxseed. The mandibular BMD increased in all three groups receiving the hydroalcoholic extract of flaxseed. Therefore, the mean difference of these groups was significant compared to the control group (table 2).

**Table 2:** Comparison of the mean differences of maxillary and mandibular BMD in flaxseed groups

			**mean differences**	pvalue
FLAX_100 mg/kg	mandible	Control	16.3	.001
		Vitd/Ca	9.75	NS^**^
		FLAX_200	-6.39	NS
		FLAX_400	3.46	NS
	maxilla	Control	16.23	NS
		Vitd/Ca	13.88	NS
		FLAX_200	-7.82	NS
		FLAX_400	1.98	NS
FLAX_200 mg/kg	mandible	Control	22.69	.001
		Vitd/Ca	16.14	.001
		FLAX_400	9.85	.047
	maxilla	Control	24.05	.001
		Vitd/Ca	21.7	.001
		FLAX_400	9.8	NS
FLAX_400 mg/kg	mandible	Control	12.84	.020
		Vitd/Ca	6.29	NS
	maxilla	Control	14.25	NS
		Vitd/Ca	11.9	NS

* : The mean difference is significant at the ≤0.05 level ^** ^ : Not Significant

The maxillary BMD increased in the groups receiving 100 and 400 mg/kg, but there was no significant difference between the two genders in these groups. The maxillary BMD was significantly higher in female rats of the group receiving 200 mg/kg flaxseed than other groups; the mean difference of this increase was significant in the group receiving 200 mg/kg flaxseed compared to the control group and the group receiving Ca/vit D (p values were 0.001 and 0.003, respectively), while the increase in BMD was not significant in male rats of the group receiving 200 flaxseed compared to the control group and the group receiving Ca/vitD. The mandibular BMD was higher in males and females of the group receiving 100 mg/kg flaxseed compared to the control group and the group receiving Ca/vit D; this increase was significant in male rats compared to controls (P=0.021). 

**Figure 3:**
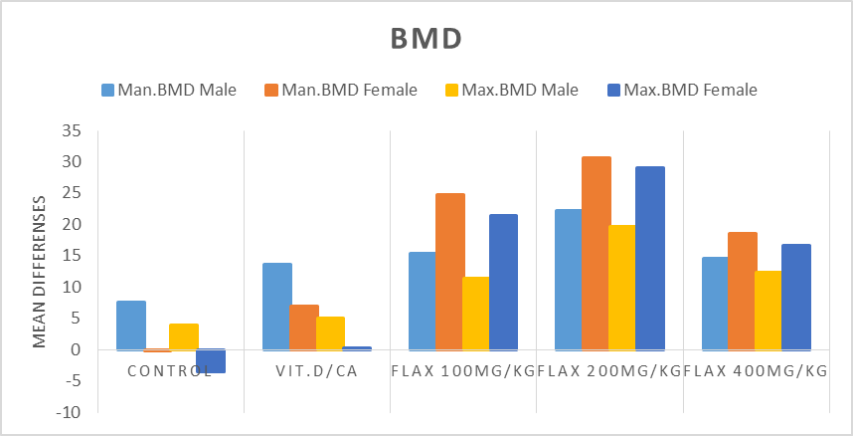
BMD changes in both jaws based on gender

The mandibular BMD was higher in males and females of the group receiving 200 mg/kg flaxseed compared to the control group and the group receiving Ca/vit D; this increase in female rats was significant compared to the control group and the group receiving Ca/vit D (p=0.001) but was not significant in male rats. In the group receiving 400 mg/kg flaxseed, the mandibular BMD of both genders increased compared to the control group and the group receiving Ca/vit D. This increase in females was significant compared to the control group (p=0.036) (figure 3).

## Discussion

The maxillary and mandibular BMD increased in all three groups receiving hydroalcoholic extract of flaxseed compared to the control group and the group receiving calcium and vitamin D and the mean difference of this increase was significant in the group receiving 200 mg/kg flaxseed compared to the control group and the group receiving Ca/vit D in the upper jaw. In the lower jaw, this increase was significant in all three groups receiving the hydroalcoholic extract of flaxseed compared to control group.

Moreover, the comparison of males and females showed that the maxillary and mandibular BMD increased in all flaxseed groups and this increase in female rats was significant in the groups receiving 200 and 400 mg/kg flaxseed but in male rats it was significant in groups receiving 100 mg/kg flaxseed. The flaxseed’s mechanism of action on bone structures is complex and includes several signal paths. Flaxseed flour has a high level of ALA (alpha-linolenic acid) and therefore, an ALA-rich diet plays a significant role in maintaining or increasing bone mass (21). On the other hand, flaxseed contains high levels of lignan phytoestrogen (8), which has both estrogenic and anti-estrogenic activities. In a study by Ward et al. on rats using flax lignans, it was concluded that continuous exposure to flax lignans increases the femur bone strength in female rats on postnatal day 50 (adolescence). However, on postnatal day 132 (adulthood), the improvement in bone strength did not constantly continue. The results of their study suggested that the positive effects of flax lignans on femur strength in male rats were limited to the adolescence period and did not continue until adulthood. Regarding the effect of flax lignans on bone mineral content (BMC), this effect was lower in the groups receiving flax lignans on postnatal days 50 and 132 compared to control group. The minimum BMC was observed on postnatal day 132 (26). Unlike the study of Ward et al., BMD increased in all groups receiving flaxseed in the present study, which might be due to the use of purified lignans in the study of Ward et al. and the use of flaxseed lignan extract in this study, which contains other active compounds that affect the bone such as ALA in addition to lignan. 

Another study by Ward et al. on male rats using purified lignans and flaxseed indicated that the femur size, BMD and bone mineral content (BMC) were not affected by anything and had no marked differences (27). Unlike the study of Ward et al., the use of flaxseed in the present study increased BMD in male rats (though this increase was only significant in 100 mg/kg flaxseed group and was not significant in other flaxseed groups), which could be due to the different dose used in this study. In this study, the effect of flaxseed extract on BMD in female rats was higher than male rats, which could be due to the positive effect of lignans in addition to other flaxseed compounds on immature rats, which indicates that female rats are more sensitive to lignans and other flaxseed extract compounds compared to male rats. Dual energy x-ray absorptiometry was used to determine the effect of flaxseed on BMD in previous studies. The present study used digital radiography, which was easily accessible and did not require the special equipment only available in hospitals or research centers.

Several studies have used flaxseed oil and have reported varied results, and it seems that the effect of ALA on bone metabolism depends on physiological and pathological conditions, since animal species, stage of development, presence or absence of osteoporosis and pathological conditions such as polycystic kidney disease, inflammatory bowel disease, obesity and insulin resistance are diverse in different studies. In general, nutrition with flaxseed oil supplement may be beneficial in increasing omega-3 unsaturated fatty acids during the bone-remodeling period, and may lead to changes in bone markers, BMD, BMC and bone strength (37-40).

The study showed that the maximum effect of the extract was achieved at 200 mg/kg dose and by increasing the dose to 400 mg/kg, BMD increased not seen in all groups receiving flaxseed compared to 200 mg/kg group, indicating that the maximum effects of flaxseed on BMD are a certain range of dose and higher doses do not increase the density. Regarding the serum levels of calcium and phosphorus as well as mean difference, they had no significant difference before and after 30 days. However, the mean difference in serum vitamin D was significantly higher in the group receiving Ca/vit D compared to the control group and the groups receiving flaxseed extract.

A number of studies have investigated the effect of flaxseed on biomarkers involved in the process of remodeling; there is now little evidence of the beneficial effects of flaxseed on bone remodeling biomarkers (41-43). In the present study, the results related to calcium, phosphorus and vitamin D levels before and after the use of flaxseed extract confirmed this.

In conclusion, it was concluded that a specified dose of flaxseed extract in adolescent rats increased BMD in both the male and female rats, and anti-estrogenic properties of flaxseed extract that had negative effects on BMD were not observed in any groups. In addition, the effect of flaxseed extract on bone density in female rats was higher than the male rats.

## References

[1] Eisman JA (1999). Genetics of osteoporosis. Endoc Rev.

[2] Teegarden D, Proulx WR, Martin BR (1995). Peak bone mass in young women. J Bone Miner Res.

[3] Johnston CC Jr, Miller JZ, Slemenda CW (1992). Calcium supplementation and increases in bone mineral density in children. N Engl J Med.

[4] Lonzer MD, Imrie R, Rogers D (1996). Effects of heredity, age, weight, puberty, activity, and calcium intake on bone mineral density in children. Clin Pediatr.

[5] Slemenda CW, Miller JZ, Hui SL, Reister TK, Johnston CC Jr (1991). Role of physical activity in the development of skeletal mass in children. J Bone Miner Res.

[6] Stoppie N, Pattijn V, Van Cleynenbreugel T (2006). Structural and radiological parameters for the characterization of jawbone. Clin Oral Implants Res.

[7] Elmadfa I (2005). Diet diversification and health promotion.

[8] Thompson LU, Robb P, Serraino M, Cheung F (1991). Mammalian lignan production from various foods. Nutr Cancer.

[9] Tham DM, Gardner CD, Haskell WL (1998). Potential health benefits of dietary phytoestrogens: a review of the clinical, epidemiological, and mechanistic evidence. J Clin Endocrinol Metab.

[10] Thompson LU (1998). Experimental studies on lignans and cancer. Bailliere's Clin Endocrinol Metab.

[11] Ward WE, Thompson LU Dietary estrogens of plant and fungal origin: occurrence and exposure. Endocrine Disruptors– Part I. Springer 2001; pp: 101-28.

[12] Ward WE, Jiang FO, Thompson LU (2000). Exposure to flaxseed or purified lignan during lactation influences rat mammary gland structures. Nutr Cancer.

[13] Clair RS (1998). Cardiovascular effects of soybean phytoestrogens. Am J Cardiol.

[14] Knight DC, Eden JA Phytoestrogens-a short review. Maturitas.

[15] Warren MP (2002). The effects of phytoestrogen supplementation in postmenopausal women. J Soc Gynecol Investig.

[16] Vanderschueren D (1996). Androgens and their role in skeletal homeostasis. Horm Res.

[17] Vanderschueren D, Van Herck E, De Coster R, Bouillon R (1996). Aromatization of androgens is important for skeletal maintenance of aged male rats. Calcif Tissue Int.

[18] Vanderschueren D, van Herck E, Nijs J (1997). Aromatase inhibition impairs skeletal modeling and decreases bone mineral density in growing male rats. Endocrinology.

[19] Adlercreutz H, Bannwart C, Wähälä K (1993). Inhibition of human aromatase by mammalian lignans and isoflavonoid phytoestrogens. J Steroid Biochem Molecular Biol.

[20] da Costa CA, da Silva PC, Ribeiro DC (2016). Effects of diet containing flaxseed flour (Linum usitatissimum) on body adiposity and bone health in young male rats. Food Funct.

[21] Dew TP, Williamson G (2013). Controlled flax interventions for the improvement of menopausal symptoms and postmenopausal bone health: a systematic review. Menopause.

[22] Lau BY, Cohen DJ, Ward WE, Ma DW (2013). Investigating the role of polyunsaturated fatty acids in bone development using animal models. Molecules.

[23] Reeves PG (1997). Components of the AIN-93 diets as improvements in the AIN-76A diet. J Nutr.

[24] da Costa CAS, da Silva PCA, Ribeiro DC (2016). Body adiposity and bone parameters of male rats from mothers fed diet containing flaxseed flour during lactation. J Dev Origins Health Dis.

[25] You L, Sheng ZY, Tang CL (2011). High cholesterol diet increases osteoporosis risk via inhibiting bone formation in rats. Acta Pharmacol Sinica.

[26] Ward WE, Yuan YV, Cheung AM, Thompson LU (2001). Exposure to purified lignan from flaxseed (Linum usitatissimum) alters bone development in female rats. Br J Nutr.

[27] Ward WE, Yuan YV, Cheung AM, Thompson LU (2001). Exposure to flaxseed and its purified lignan reduces bone strength in young but not older male rats. J Toxicol Environmental Health A.

[28] Dural S, Ozbek M, Kanli A (2005). Evaluation of mandibular bone density to predict osteoporosis in adolescents with constitutional delayed growth. Saudi Med J.

[29] Nackaerts O, Jacobs R, Horner K (2007). Bone density measurements in intra-oral radiographs. Clin Oral Investig.

[30] White SC, Atchison KA, Gornbein JA (2005). Change in mandibular trabecular pattern and hip fracture rate in elderly women. Dentomaxillofac Radiol.

[31] Yang J, Chiou R, Ruprecht A (2002). A new device for measuring density of jaw bones. Dentomaxillofac Radiol.

[32] Geraets WG, van der Stelt PF (2000). Fractal properties of bone. Dentomaxillofac Radiol.

[33] Lindh C, Petersson A, Rohlin M (1996). Assessment of the trabecular pattern before endosseous implant treatment: diagnostic outcome of periapical radiography in the mandible. Oral Surg Oral Med Oral Pathol Oral Radiol Endod.

[34] Seyedmajidi M, Rabiee S, Haghanifar S (2015). Histopathological, histomorphometrical, and radiographical evaluation of injectable glass-ceramic-chitosan nanocomposite in bone reconstruction of rat. Int J Biomater.

[35] Seyedmajidi M, Haghanifar S, Hajian-Tilaki K, Seyedmajidi S (2018). Histopathological, histomorphometrical, and radiological evaluations of hydroxyapatite/bioactive glass and fluorapatite/bioactive glass nanocomposite foams as cell scaffolds in rat tibia: an in vivo study. Biomed Mater.

[36] Ebrahimi Vosta Kalaee S, Talebi Mazraeh Shahi A, Naseri M (2014). Anti-inflammation effect of alcoholic extract of linum. J Babol Univ Med Sci.

[37] Cohen SL, Ward WE (2005). Ward, flaxseed oil and bone development in growing male and female mice. J Toxicol Environ Health A.

[38] Farmer C, Petit HV, Weiler H, Capuco AV (2007). Effects of dietary supplementation with flax during prepuberty on fatty acid profile, mammogenesis, and bone resorption in gilts. J Animal Sci.

[39] Li Y, Greiner RS, Salem N Jr, Watkins BA (2003). Impact of dietary n− 3 FA deficiency on rat bone tissue FA composition. Lipids.

[40] Weiler HA, Fitzpatrick-Wong SC (2002). Modulation of essential (n-6):(n-3) fatty acid ratios alters fatty acid status but not bone mass in piglets. J Nutr.

[41] Arjmandi BH, Juma S, Lucas EA (1998). Flaxseed supplementation positively influences bone metabolism in postmenopausal women. JANA.

[42] Dalais FS, Rice GE, Wahlqvist ML (1998). Effects of dietary phytoestrogens in postmenopausal women. Climacteric.

[43] Lucas EA, Wild RD, Hammond LJ (2002). Flaxseed improves lipid profile without altering biomarkers of bone metabolism in postmenopausal women. J Clin Endocrinol Metabolism.

